# Genomic characterization of a novel *Hepatovirus* identified in Maranhão state, Brazil

**DOI:** 10.1038/s41598-024-58171-y

**Published:** 2024-04-05

**Authors:** Walna Micaelle de Moraes Pires, Ana Cecília Ribeiro Cruz, Alex Junior Souza de Souza, Sandro Patroca Silva, Taciana Fernandes Souza Barbosa Coelho, Daniel Damous Dias, José Wilson Rosa Júnior, Samira Brito Mendes, Elmary da Costa Fraga, Maria Claudene Barros, Iracilda Sampaio

**Affiliations:** 1 Graduate Program in Genetics and Molecular Biology, Universidade Federal do Pará-UFPA, Belém, Pará 66075-110 Brazil; 2 Department of Arbovirology and Hemorrhagic Fevers, Instituto Evandro Chagas IEC/SVS/MS, Ananindeua, Pará 67030-000 Brazil; 3 Department of Pathology, School of Veterinary Medicine and Animal Science, University of São Paulo (USP), São Paulo, Brazil; 4 Laboratory of Medical Entomology, Instituto Evandro Chagas IEC/SVS/MS, Ananindeua, Pará 67030-000 Brazil; 5 Graduate Program in Biodiversity and Biotechnology-Bionorte Network, Laboratory of Genetics and Molecular Biology, Universidade Estadual do Maranhão, São Luís, Maranhão 65055-310 Brazil; 6 Laboratory of Genetics and Molecular Biology-GENBIMOL, Universidade Estadual Do Maranhão-Campus Caxias, Caxias, Maranhão 65604-380 Brazil; 7 Laboratory of Evolution, Institute of Coastal Studies, Universidade Federal do Pará-UFPA-UFPA, Bragança, Pará 68600-000 Brazil

**Keywords:** Cell biology, Computational biology and bioinformatics, Genetics, Microbiology, Molecular biology, Health care

## Abstract

Bats are efficient reservoirs of a number of viruses with zoonotic potential, and are involved directly in the transmission cycle of many zoonoses. In the present study, which is part of a larger project that is documenting the viromes of the bat species found in the Mid-North states of Maranhão and Piauí, we analyzed 16 pooled samples obtained from four species of bat of the genus *Artibeus—Artibeus obscurus, Artibeus cinereus, Artibeus lituratus* and *Artibeus planirostris*. We describe and identify a *Hepatoviru*s, denominated *Hepatovirus H* isolate *sotense*, which was found in a pool of internal organs (liver and lungs) extracted from a specimen of *A. planirostris*, a frugivorous bat, collected in the Cerrado biome of Maranhão state. This material was analyzed using new generation sequencing, which produced a contig of 7390 nucleotides and presented a degree of identity with a number of existing *Hepatovirus* sequences available for bats (amino acid identity of 61.5% with Bat *hepatovirus* C of *Miniopterus* cf. *manavi*, 66.6% with Bat *hepatovirus* G of *Coleura afra*, 67.4% with *Hepatovirus G2* of *Rhinolophus landeri*, and 75.3% with *Hepatovirus H2* of *Rhinolophus landeri*). The analysis of the functional domains of this contig confirmed a pattern consistent with the characteristics of the genus *Hepatovirus* (*Picornaviridae*). In the phylogenetic tree with several other *Hepatovirus* species, this genome also grouped in a monophyletic clade with *Hepatovirus H* (HepV-H1; HepV-H2, and HepV-H3) albeit on an external branch, which suggests that it may be a distinct genotype within this species. This is the first isolate of *Hepatovirus H* identified in bats from South America, and represents an important discovery, given that most studies of viruses associated with bats in the state of Maranhão have focused on the family *Rhabdoviridae.*

Bats are placental mammals of the order Chiroptera, with at least 1384 recognized species^1^, of which, approximately 15% occur in Brazil^2^. In addition to their ecological importance^3^, bats are also considered to be prominent virus hosts^4^. Pandemics caused by zoonoses reinforce the need for the monitoring of new viruses found in wild hosts, in particular, in bats and rodents, which are the mammals that harbor the greatest diversity of potentially zoonotic viral species^5^.

A total of 17 viral families have been identified in Brazilian bats, although only a small proportion (23.75%) have been the focus of research over the past 20 years^6^. The order Picornavirales—Pisoniviricetes: Pisuviricota^7^ is considered to be the largest and most diverse of the positive strand RNA viruses. The family *Picornaviridae* includes 158 species distributed in 68 genera^8^, which are capable of infecting an enormous range of vertebrate hosts. These viruses include some important pathogens, such as the poliovirus, and the viruses that cause hepatitis A and foot-and-mouth disease^9^.

A number of distantly-related hepatotropic viruses have recently been discovered in bats, rodents, and other small mammals^10^. These viruses are related phylogenetically, sharing antigenic determinants with the human hepatitis virus A^11^. Bats are known to have played an important role in the emergence and evolution of many viruses, which reinforces the fundamental need to investigate the virome of these animals. It appears reasonable to assume that the diversity of the viruses of the genus *Hepatovirus* has yet to be fully understood^12^. Records from the DBatVir platform indicate that 321 picornavirus sequences have been deposited in this database, of which, 48 refer to the genus *Hepatovirus*, although the bat genus *Artibeus* was not cited in any of these records^13^.

The present study provides the description and identification of the genome of a new hepatovirus, *denominated Hepatovirus H* isolate *sotense*, retrieved from a specimen of the flat-faced fruit-eating bat, *Artibeus planirostris*, collected in the Maranhão State, Mid-North region of Brazil.

## Results

### Sampling

The material analyzed here consisted of 16 samples (pools) of internal organs (liver and lungs) obtained from four bat species of the genus *Artibeus* from different localities in the Mid-North region of Brazil—*Artibeus obscurus* (Pool 2—Timon and Pool 8—Carolina), *Artibeus cinereus* (Pool 1—Timon and Pool 3—Caxias), *Artibeus lituratus* (Pool 4—Gurupi, Pool 6—Timon, and Pool 10—Carolina), and *A. planirostris* (Pool 5—São João do Sóter, Pool 7—Timon, Pool 9, 11–16—Carolina). For the sample of the species *A. planirostris* (Pool 5—São João do Sóter), the genome of a new hepatovirus, called *Hepatovirus H* isolate *sotense*, was described and identified.

### Sequencing and bioinformatic analyses

**Figure 1:**
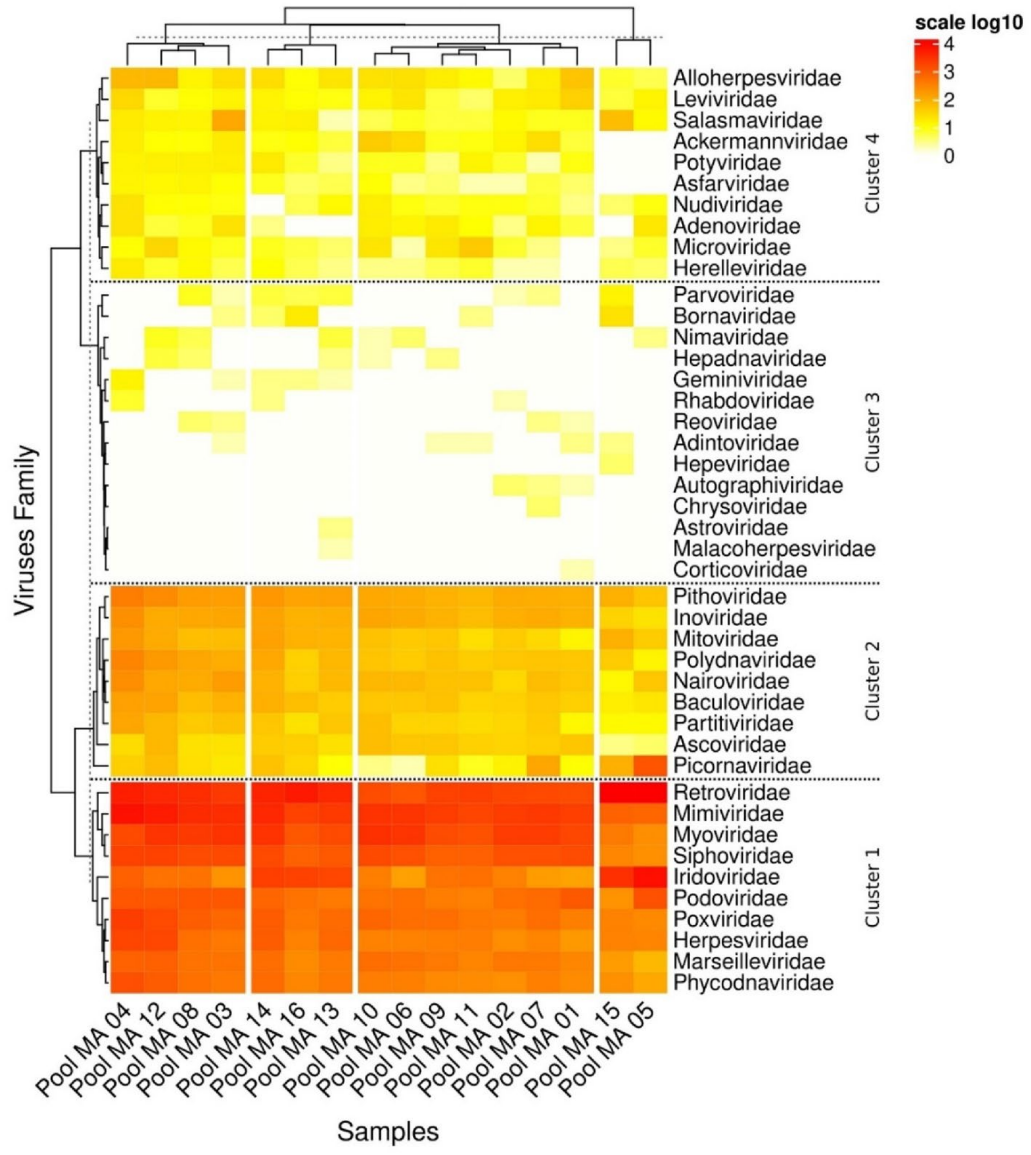
Heat map showing the distribution of the reads for the different viral families. The figure shows the viral families that were detected. The number of reads is indicated by the color scale, which ranges from light yellow, for the families with the smallest number of reads, to bright red, which indicates the largest number of reads. The *Retroviridae, Mimiviridae, Siphoviridae*, and *Myoviridae* were the most frequent viral families.

The genomic libraries (cDNA) were constructed based on a total of 54,681,026 million read, on average, with these reads being used to compile a de novo arrangement. The taxonomic classification programs estimated 54,68,926 microbial reads, with the lowest number—45,919—being recorded for viruses. When visualized in the MEGAN software, the alignment indicated considerable viral diversity, with reads corresponding to 39 partial sequences related to RNA viruses, belonging principally to the families *Retroviridae, Mimiviridae, Siphoviridae* and *Myoviridae*, as shown in the heat map (Fig. 1). The distribution of these families in the heat map indicates the presence of the complete *Hepatovirus* genome in *pool* 5, which will be analyzed and described in detail below.

### High-throughput sequencing

The genome of 7390 nucleotides (nt) has untranslated regions (UTRs) of 737 nt and 64 nt at the 5' and 3' ends, respectively. The mean genome coverage was 26×, with a GC content of 36.6%. The analysis of the open reading frame matrix identified a protein with 2000 predicted amino acids and a molecular weight of 248,690 kDa. This genome has been deposited in the GenBank public database under the name *Hepatovirus H* isolate *sotense* (Accession number: OR367421), with the codes BioProject PRJNA947063, BioSample: SAMN36696548, Sequence Read Archive: SRR24657753. The raw metagenomic data are available on the code OR367421 for *Hepatovirus H* isolate *sotense*, in the link (https://www.ncbi.nlm.nih.gov/nuccore/OR367421). The BlastX result indicated an identity of 76.8% with *Hepatovirus H2* (Accession number: KT452714), isolated from a bat (*Eidolon helvum*) in Ghana in 2010.

### Phylogenetic and similarity analysis

**Figure 2:**
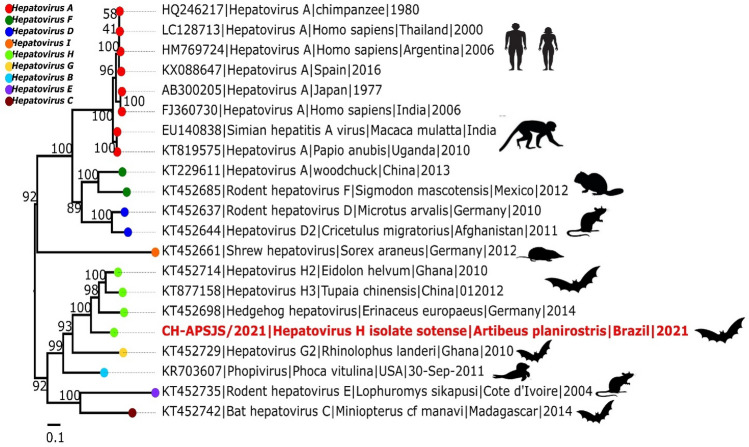
Phylogenetic neighbor-joining tree of the *Hepatovirus* species based on their amino acid sequences, compiled in IG-TREE com 1000 bootstrap replicates. The sequence obtained in the present study is highlighted in italics, with two asterisks.

The phylogenetic analyses revealed two major clades formed by *Hepatovirus A, F, D, I* and *Hepatovirus H, G, B, E, C* (Fig. 2). *Hepatovirus H* isolate *sotense* was assigned to a monophyletic clade within the *Hepatovirus H* species, however it occupied the most basal branch of the clade, with high (100%) bootstrap support.

A matrix was compiled which grouped the hepatoviruses that are closest to *H. H* isolate *sotense*, which showed that this species was most similar to the sequence of *Hepatovirus H2* (72.7% nt and 76.8% aa) and *Bat Hepatovirus* G (64.9% nt and 66.5% aa). The values found here indicate clearly that, although *H. H* isolate *sotense* is closest to hepatoviruses H2 but, it is clearly different from it, which reinforces its status as a new genotype of *Hepatovirus H* species (Table 1).

**Table 1:** Comparisons of the identity of *Hepatovirus H isolate sotense* sequences with those that showed greatest proximity in the phylogenetic grouping.

	Samples	1	2	3	4	5	6	7	8	9	10
1	*Hedgehog hepatovirus H* KT452695		98.4	79.0	78.0	75.1	65.6	65.4	66.1	57.9	54.9
2	*Hedgehog hepatovirus H* KT452698	90.5		79.1	77.8	74.9	65.7	65.3	65.7	57.8	54.9
3	*Hepatovirus H2* KT452714	73.7	74.0		83.8	76.8	66.0	66.3	66.3	59.0	55.2
4	*Tupaia hepatovirus H3* KT877158	71.9	71.6	75.3		75.8	65.1	65.2	66.3	58.5	55.1
5	*Hepatovirus H* isolate *sotense H* OR367421	71.9	71.8	72.7	71.3		66.7	66.5	66.0	58,4	55.2
6	*Hepatovirus G2* KT452729	64.7	64.5	64.7	64.0	65.9		73.3	64.0	55.7	53.5
7	*Bat hepatovirus G* BUO2BF86	64.3	64.1	65.0	63.5	64.9	70.6		62.8	57.1	53.0
8	*Phopivirus B* KR703607	66.0	66.0	66.4	64.9	65.5	65.6	64.1		58,2	55.0
9	*Bat hepatovirus C* SMG18520	61.7	61.1	61.9	61.0	61.0	59.5	59.5	61.5		58.6
10	*Rodent hepatovirus E* CIV459	59.2	59.3	59.6	58.6	58.6	57.7	56.2	58.3	60.6	

The values below the diagonal refer to the nucleotide sequences, and those above the diagonal refer to the amino acid sequences.

Given the overall pattern found in the phylogenetic tree and this matrix, a new analysis of similarity was run to verify the level of differentiation among the *Hepatovirus* species, correlating the amino acid and nucleotide sequences of the ORF. This analysis indicated that the sequence of *Hepatovirus H* isolate *sotense* is closely related to those of the other viruses of genus, but is also clearly distinct from them. The identity (differences per site between the sequences) of the species ranged from 55.5 to 99.7% in the case of the amino acids and 50.0–99.9% for the nucleotides. Considering only the clade that unites the *H* species in the phylogenetic tree, the identity of the amino acids was 71.3–98.0%, while that of the nucleotides was 74.9–99.6% (Supplementary Table 1). When only the P1 region of the polyprotein was considered in this comparison, the values were 60.5–99.9% and 64.0–99.9%, respectively. In the specific case of *Hepatovirus H*, the ranges were 85.0–99.6% and 75.0–96.8%, respectively (Supplementary Table 2). One last matrix was compiled, considering the 2C+3CD regions of the polyprotein, and once again, the same pattern was found, with an identity of 52.0–99.8% for the nucleotides and 57.0–99.6% for the amino acids, considering all the heptoviruses, and 77.6–99.5% and 71.5–98.1%, respectively, for *Hepatovirus H* (Supplementary Table 3).

### Genomic characterization

The ordination of the sequences revealed the classic organization of the genome of the members of the genus *Hepatovirus*, which confirms the classification *of Hepatovirus H* isolate *sotense* as a picornavirus. The structure identified in this analysis indicated the presence of a single ORF, which codifies a polyprotein. The viral polyprotein is divided into three regions: P1, P2, and P3. The P1 region encodes the viral capsid proteins (VP4-VP2-VP3-VP1), whilst the P2 and P3 regions encode proteins involved in protein processing (2A^pro^, 3C^pro^ and 3CD^pro^) and genome replication (2B, 2C, 3AB, 3B^VPg^, 3CD^pro^, 3D^pol^)^14^. The proteolytic cleavage map was derived from the alignment by analogy with the other hepatovirus sequences that grouped with *Hepatovirus H* isolate *sotense* in the analysis presented here. While the organization of the genome of *Hepatovirus H* isolate *sotense* is typical of that of other hepatoviruses, it was differentiated from the other members of the genus *Hepatovirus*. In particular, there is a high level of variation in the specific protein-codifying regions of the different species of the genus. As can be seen here, *Hepatovirus H* isolate *sotense* has a poorly-conserved region downstream from VP1, in comparison with the other hepatoviruses analyzed here (Supplementary Fig. 2), which is symptomatic of the differentiation of the species of this genus. The mean level of similarity of the proteins found in the different hepatovirus species, including *Hepatovirus H* isolate *sotense*, is shown in Table 1 (see also Fig. 2). The analysis of the 10 sequences (*Hepatovirus H* isolate *sotense, Phopivirus; Hedgehog hepatovirus* Igel8Erieur2014; *Hedgehog hepatovirus; Hepatovirus H2; Hepatovirus G2; Bat hepatovirus* BUO2BF86Colafr2010; *Rodent hepatovirus* CIV459Lopsik2004; *Bat hepatovirus* SMG18520Minmav2014; *Tupaia hepatovirus* A) showed that they were, in general, less similar to one another than those of the five sequences (*Hepatovirus H* isolate *sotense; Hedgehog hepatovirus; Hedgehog hepatovirus Igel8Erieur2014; Hepatovirus H2; Tupaia hepatovirus A*). This indicates that the species of the latter group are more closely-related, even though they are clearly differentiated.

**Figure 3:**
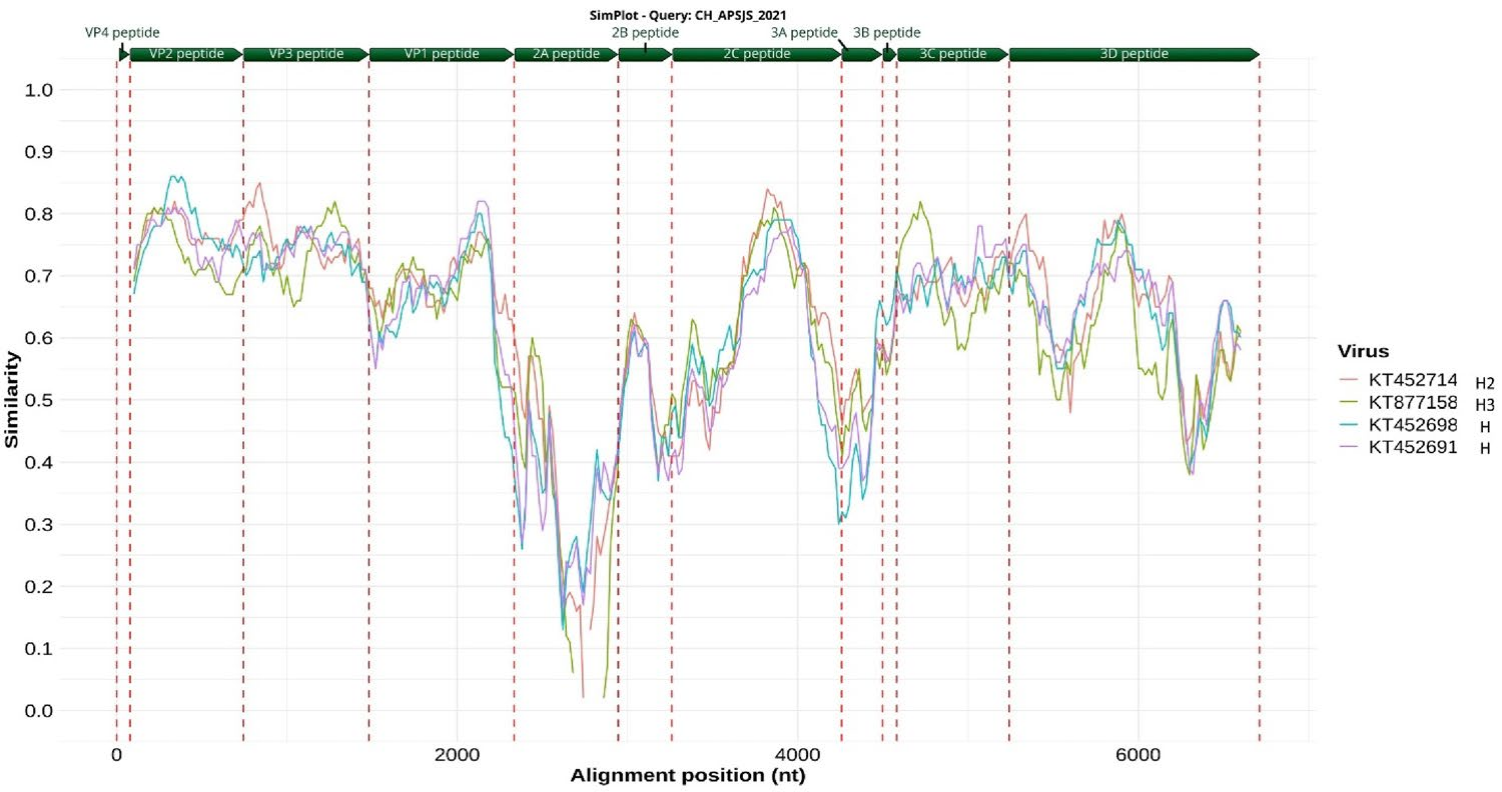
Similarity analysis using SimPlot. The figure illustrates the SimPlot analysis of the query sequence of *Hepatovirus H* isolate *sotense* genotype CH-APSJS/2021 (NCBI ID OR367421). Identification of all proteins is presented at the top of the chart. The analysis employed the following parameters: Window: 200 bp, Step: 20 bp, GapStrip: On, Kimura (2-parameter), T/t: 2.0. The alignment positions indicated in nucleotides (nt)*.*

The nucleotide sequences of the four most closely related *Hepatovirus* genotypes in the phylogenetic tree (*Hedgehog hepatovirus,* KT452691; *Hedgehog hepatovirus* Igel8Erieur2014, KT452698; *Hepatovirus H2,* KT452714; and *Tupaia hepatovirus A*, KT877158) were analyzed using the Simplot software, with *Hepatovirus H* isolate *sotense* serving as the reference sequence. The similarity plot resulting from this analysis (Fig. 3) indicates that a substantial similarity, with mean percentages in the upper 70%, in the VP1 region of *Hepatovirus H* isolate *sotense* was observed within this species. Subsequently, the P3 region exhibited relatively lower similarity, while the lowest similarity, around 70%, was observed in the P2 region.

**Figure 4:**
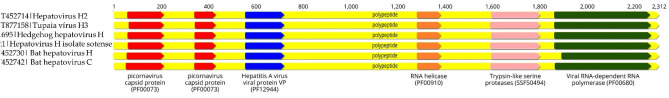
Demonstration of the different functional domains of the polyprotein from various viruses belonging to the *Hepatovirus* species. The yellow arrow corresponds to the coding region of the polyprotein. The different domains are depicted in colors according to the identified protein, and their respective protein codes are in parentheses.

The analysis of the functional domains indicated that *Hepatovirus H* isolate *sotense* shares its pattern of genomic organization with the samples that were most closely related in the phylogenetic analysis. This further confirms the taxonomic status of this species within the genus *Hepatovirus* (Fig. 4).

The results of the present study are part of a larger project that is describing the viromes of different species of bat. In general, the isolation of hepatoviruses, and other hepatotrophic viruses, is a major challenge, even considering the most common cell lines. In the specific case of bats, few studies have been able to isolate effectively the hepatoviruses of these animals, although, in the future, when the techniques have been fully standardized, the target viruses will be isolated from the FRhK-4 cells of all the samples in which some type of viral infection has been confirmed by immunofluorescence.

In the specific case of the Artibeus species analyzed here, samples of the lung, liver, heart, spleen, intestine, brain, kidney, and blood were examined by RT-PCR to determine the presence of viruses in specific organs.

## Discussion

The results of the present study indicate the discovery of a novel isolate of *Hepatovirus H*, in the genus *Hepatovirus*. These hepatoviruses are known to include many pathogens of both humans and other vertebrates, causing diseases that affect mainly the liver^15^. While many new *Hepatovirus* species have been found recently using new generation sequencing, the pathogenicity of most of these viruses is still relatively poorly understood^16^. As chiropterans are known to act as reservoirs of an ample variety of viral agents^5^, it will be extremely important to identify and describe the viromes of the different species of this mammalian order, to provide an ever more solid foundation for further research in epidemiology and public health. As far as we know, this is the first report of a *Hepatovirus* in bats from South America, considering that the only previous description was from Central America (Costa Rica), with the virus occurring in *Natalus lanatus*, with sequences being obtained from partial samples of two isolates, CR10401 (KT452716.1) and CR10366 (KT452715.1)^16^. Furthermore, according to data from the DBatVir Platform researched in November 2023^13^, there are no records of *Hepatovirus* in any of the species of the genus *Artibeus*, with the first being described here.

A number of new *Hepatovirus* species have been described in recent years, resulting in an exponential growth in the known diversity of picornaviruses, as well as an ample variety of hosts, distributed throughout the world, that can be parasitized by these viruses^16^. One study of small mammals of three different orders^16^ identified 13 new *Hepatovirus* species, including four that infect bats. Two other new species of *Hepatovirus* have been identified from specimens of seals^17^, and one new species was described in a study of marmots^18^. In the specific case of the Bat *Hepatovirus*, a recent study^19^ identified a new species in *Hipposideros armiger* from China, while a second study^20^ recorded a new species in *Eptesicus fuscus* in the United States. It is worth noting that of all these species, *Hepatovirus H* isolate *sotense* obtained in the present study was shown to be most closely related to *Hepatovirus G.* Based on the data from the International Committee on Taxonomy of Viruses (ICTV), only Hepatovirus A is capable of causing natural infectious hepatitis in humans, and experimental infection in other primatas, although persistent infection does not occur in vivo, and the viruses are not associated with chronic hepatitis. Few biological data are available on the other hepatoviruses, however, or the clinical manifestations they cause in their hosts^14^. While there has been a considerable recent increase in the number of species, these new hepatoviruses have been assigned clearly to the respective genus, given the greater distances that separate them from the genus *Tremovirus*, which represents the closest picornavirid lineage, as well as the criteria adopted by the ICTV for the classification of picornaviruses^9^.

It is important to note here that, for the ICTV to accept the proposal of these new species, for the classification of the new hepatovirus variants, it was necessary to provide complete genome sequences, and demonstrate their clear separation in the phylogenetic analyses^21^. In the present study, it was demonstrated clearly that the proposed taxon is a new genotype belonging to *Hepatovirus H*, here named *Hepatovirus H* isolate *sotense*, taking the findings of the present study into consideration. These results showed that the isolate was less than 30% divergent in the polyprotein amino acid sequence, less than 30% divergent in the P1 amino acid sequence, less than 30% divergent in the 2C + 3CD amino acid sequence, and shared a common genomic organization with the other species analyzed. These criteria were adopted to define the members of a species in the genus *Hepatovirus*.

The complexity of the genealogy of the hepatoviruses may be at least partly a result of the frequent changes in host that occurred in the distant past of the evolutionary history of the viruses^22^. The phylogeny presented here confirms this pattern, given that the viruses tended to group according to their different hosts, as shown clearly by the fact that the most basal clade is composed of the viruses of rodent, bat (including *A. planirostris*), hedgehog, tree shrew, and seal hosts, with high levels of statistical support. This analysis clearly demonstrates that *Hepatovirus H* isolate *sotense* belongs to the genus *Hepatovirus*, more specifically to species *H*, given that the statistical values support this claim. However, it is a new strain, as it did not form a monophyletic cluster with the other representatives of the species.

The only clearly defined clade in the phylogenetic tree is that of the human *Hepatovirus A* (HAV). Recent studies indicate that the evolutionary history of the HAVs is distinct from that of the other hepatoviruses described up to now, with differences in the nucleotide sequence of the genome and the structure of the capsid, characteristics that are shared with a primitive insect virus^23^. These viruses are also capable of releasing infected cells that appear to be enclosed in a membrane, to become virions, which are almost completely enveloped, a unique trait among the picornaviruses. In addition, cells infected by HAV may release vesicles coated with a membrane that contains a virion, resulting in unusual virions enveloped in a membrane, which are not observed in other picornaviruses^24^.

Overall, then, the sum of the evidence presented here supports the recognition of a new *Hepatovirus H* virus, denominated *Hepatovirus H* isolate *sotense*, which was isolated from the internal organ sample (liver and lung) of a specimen of the fruit-eating bat, *Artibeus planirostris*, and was clearly a picornavirus with genomic structure typical of the hepatoviruses. This is an important discovery, not least because this is the first study of its kind from the Brazilian state of Maranhão, but also because most previous studies of viruses associated with bats in this state have focused on species of the family *Rhabdoviridae.* It is important to note here that all the specimens collected for the present study tested negative for the rabies virus. It is important to note here that previous studies^22^ have shown that hepatoviruses are capable of swapping hosts. In this case, the species described here deserves further consideration, given that among the species of *Hepatovirus* that infect bats (*C, E, G*, and *H*), species *H* is the one that has the ability to infect different vertebrate species, in addition to bats, and is therefore more prone to the occurrence of mutations in its genome, and thus demands attention from the state’s epidemiological monitoring agencies. It is also important to note, in this context, that the specimen analyzed here did not present any clinical manifestations of the disease, such as an inflamed liver, or other signs of ongoing infection, which is typical of many viral pathogens in bats, and hampers the diagnosis of infection based solely on the behavior of the individual.

## Conclusions

While the number of studies of viral diversity in Brazil has increased steadily in recent decades, and in particular following the COVID-19 pandemic, research in this field is still incipient, especially in the state of Maranhão. Studies of the viruses of wild animals are important, not only to catalog this diversity, but also to identify species (as in the case of the hepatoviruses) that are potentially pathogenic for animals and humans. This knowledge will be fundamental for the elaboration of effective public health strategies, and the development of measures for the prevention or eventual mitigation of outbreaks, given that epidemiological studies are still not focused on the origins of the disease, that is, they tend to focus on outbreaks, rather than prevention. This is worrying, from an epidemiological perspective, and means that the effective prevention and control of diseases will depend on a more systematic approach, linking the health of humans, animals, and the environment. Overall, the findings of the present study provide important new insights into the diversity, hosts, and geographic distribution of the genus *Hepatovirus,* as the range of occurrence of this virus in bats increases to South America.

## Materials and methods

### Ethics statement

Field procedures and protocols for trapping, euthanasia and tissue processing, conforming to the guidelines of the National Council for the Control of Animal Experimentation (CONCEA), aiming to comply with the animal welfare standards of CFMV Resolution No. 1000/2012 and Law 11,794/2006. The collection of specimens was authorized by federal license ICMBIO/SISBIO No. 68047-2. The animals were sedated with a combination of ketamine and xylazine intramuscularly and subsequently euthanized with an overdose of lidocaine in the foramen magnum. The collection of the animal viscera (a pool of lung and liver) was carried out by veterinarians, in compliance with biosafety standards. The samples collected were stored in cryogenic vials, which were duly identified and stored in liquid nitrogen for later forwarding to the Arbovirology and Hemorrhagic Fevers laboratory of the Instituto Evandro Chagas (IEC), where the samples were handled in a laminar flow cabinet at biosafety level 2 by personnel trained in the use of personal protective equipment, with viscera maceration taking place in the NB3 laboratory. The Ethics Committee Council of CEMAVE and SISBIO reviewed and approved the field protocols and experimental procedures through authorization protocol 025/2019.

### Sample collection area

**Figure 5:**
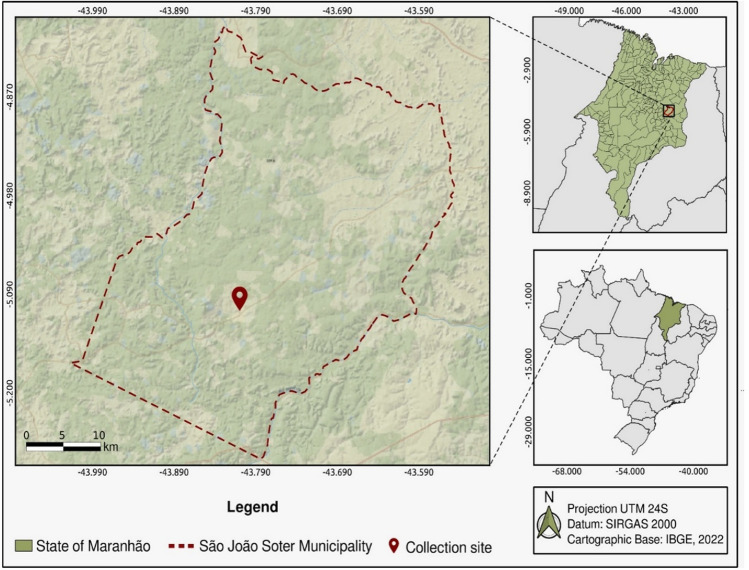
*Artibeus planirostris* collecting locality in the Brazilian state of Maranhão. Map produced in QGIS 3.22.4

The material analyzed here consisted of 16 samples (*pools*) of viscera (liver and lungs) obtained from four bat species of the genus *Artibeus—A. obscurus* (*Pool* 2*—*Timon and *Pool* 8*—*Carolina), *A. cinereus* (*Pool* 1*—*Timon and Pool 3*—*Caxias), *A. lituratus* (*Pool* 4*—*Gurupi, *Pool* 6*—*Timon, and *Pool* 10*—*Carolina), and *A. planirostris* (*Pool* 5*—*São João do Sóter, *Pool* 7*—*Timon, *Pool* 9, 11*–*16*—*Carolina). Each *pool* contained up to five tissue samples, depending on the species and collecting locality. The specimen of *A. planirostris* analyzed in the present study was collected in a mist net on 28-Oct-2021 in the municipality of São João do Sóter (Fig. 5), during the capture of specimens in areas of the Amazon and Cerrado biomes in the northern Brazilian state of Maranhão, as part of a project investigating viral pathogens in bats of the genus *Artibeus*. The sample from which the complete sequence of *Hepatovirus H* isolate *sotense* was obtained from *pool* 5 corresponds to a young adult male *A. planirostris*, with a body weight of 29 g and no apparent symptoms of any zoonosis known to affect bats, such as excessive salivation, lethargic or aggressive behavior, and alterations of the size or coloration of the internal organs.

### Metagenomic sequencing

For analysis, the genetic material was extracted from combined samples of liver and lung tissue, which were macerated together with 1 mL of the TRIzol™ Plus RNA Purification kit and a 5-mm diameter steel sphere by agitation in a TissueLyser II. The extracted RNA was purified and isolated using a PureLink RNA Mini kit, following the manufacturer’s instructions. The simple and double-strand cDNA was synthesized using the SuperScript^TM^ VILO^TM^ Master Mix (Thermo Fischer Scientific, Waltham, MA, USA) and NEBNext^®^ mRNA Second Strand Synthesis Module (New England BioLabs, Ipswich, MA, USA) kits, respectively.

### Genomic data analysis

The genomic library was prepared following the guidelines of the Nextera XT DNA kit, and sequenced in the NextSeq 500 platform (Illumina, Inc) using the NextSeq 500/550 High Output kit v2.5 (300 cycles) and the paired-end approach, following the manufacturer’s recommendations.

The quality of the raw data was evaluated by first using Fastp v0.23.2^25^ to remove the short reads (less than 50 nt), fragments of adaptors and reads with indeterminate bases (reads with more than 10% of the N).

The ribosomal RNA was then removed in SortMeRNA v2.1^26^. The processed reads were then compared with the non-redundant (nr) amino acid sequence database of the NCBI using DIAMOND v2.1.7^27^ with an e-value of 1e−4. The DIAMOND output file was visualized in MEGAN6^28^.

The reads were also mounted using the de novo approach in SPAdes v3.13.1^29^ and Megahit v1.2.9^30^, with k-mer values of 21, 33, 55 and 77 and of 21, 31, 41, 51, 61, 71, 81, 91, and 99. The grouped contigs (scaffolds) were mapped in DIAMOND^27^ with the same parameters used for the reads, and then inspected in MEGAN6^17^. The contigs that were similar to related viral sequences were evaluated in Geneious v9.1.8^31^.

### Phylogenetic analyses

The phylogenetic inference was based on the amino acid sequences of the different *Hepatovirus* strains available in the NCBI database, using the protein-codifying region. The datasets generated here were submitted, together with the present sample, to multiple sequence alignment (MSA) in Mafft v.7^32^. The result of this alignment was inspected manually, and corrected when necessary, in Geneious v9.1.8^31^.

The aligned dataset was initially analyzed to identify the best nucleotide substitution model before constructing the phylogenetic trees using the Maximum Likelihood (ML) method^33^. Both these analyses were run in IQ-TREE v1.6.12^34^.

The ML analysis was complemented with a bootstrap test, based on 1000 replicates, to determine the reliability of the groups identified in the analysis^35^. The phylogeny was visualized in FigTree v1.4.4^36^. It was decided not to use a root sequence for the dataset, but rather a mid-point rooting approach, which is available in the phylogeny visualization program. Following the evaluation and edition of the phylogeny, an “.svg” (Scalable Vector Graphics) file was generated for the edition and adjustment of the image in Inkscape v1.2^37^. All procedures were were conducted in accordance with the relevant guidelines and regulations.

### Supplementary Information

Supplementary Figures.

Supplementary Table S1.

Supplementary Table S2.

Supplementary Table S3.

## Data Availability

GenBank accession numbers. The raw metagenomic data are available on the code OR367421 for Hepatovirus H isolate sotense, BioProject PRJNA947063, BioSample: SAMN36696548, Sequence Read Archive: SRR24657753 in the link https://www.ncbi.nlm.nih.gov/nuccore/OR367421. The data are simultaneously made available to other INSDC databases, the European Nucleotide Archive (ENA) and the DNA Data Bank of Japan (DDBJ).
