# Supplementary material for: Genomic characterization of a novel Hepatovirus identified in Maranhão state, Brazil
Source: Sci Rep. 2024 Apr 5;14:7981. doi: 10.1038/s41598-024-58171-y (PMC10995186; doi:10.1038/s41598-024-58171-y)

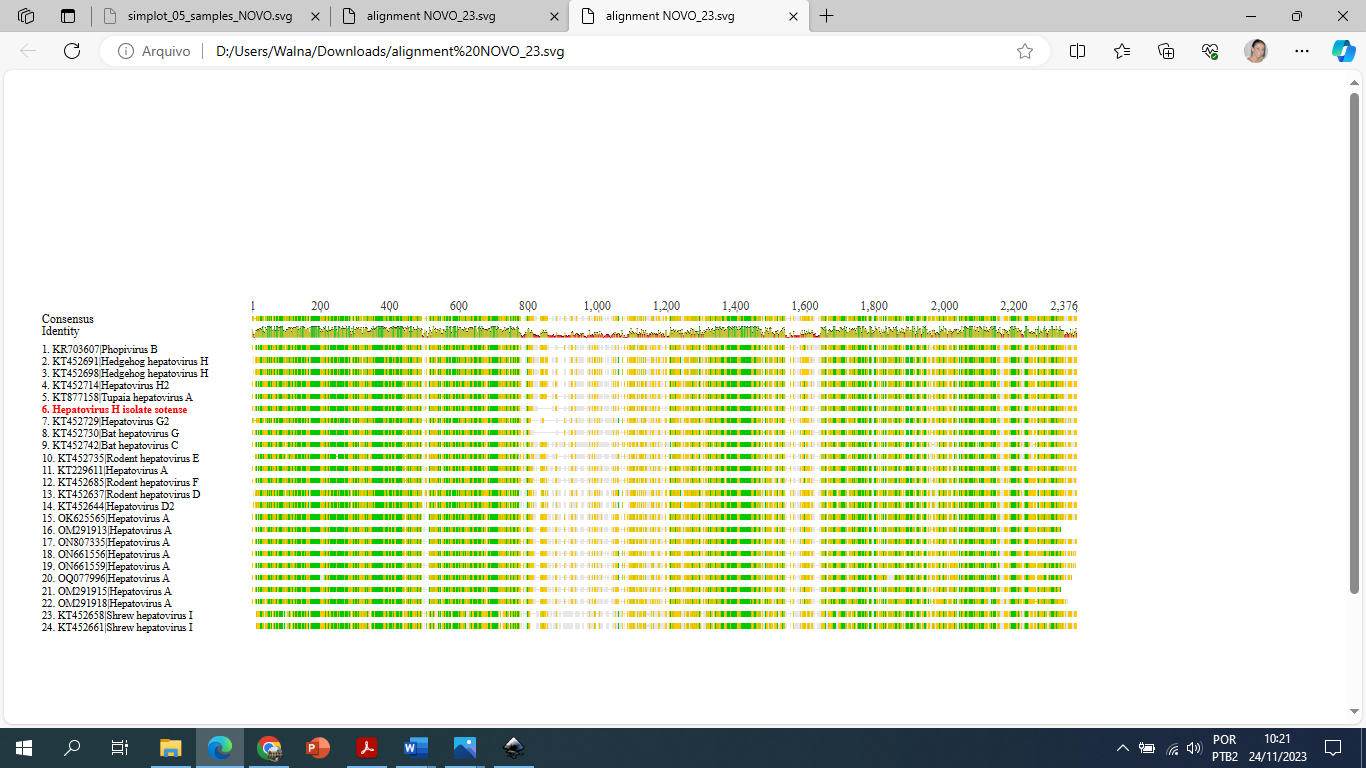


**Fig. S1**. Comparison of the genomic sequence of *Hepatovirus H isolate sotense* with those of hepatoviruses described in previous studies. The bases shaded dark green are 100% similar, the light green bases are 80–100% similar, and the yellow bases, 60–80% similar, while white indicates a similarity of less than 60%.

**Fig. S2**. Demonstration of the different functional domains of the polyprotein of different viruses belonging to the genus *Hepatovirus*. The different domains are shown in green.


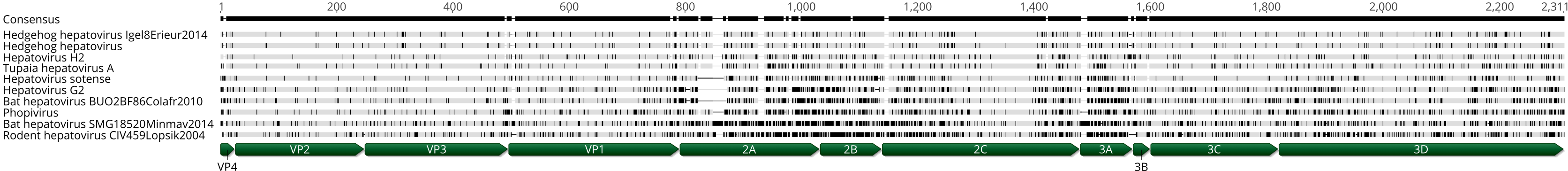

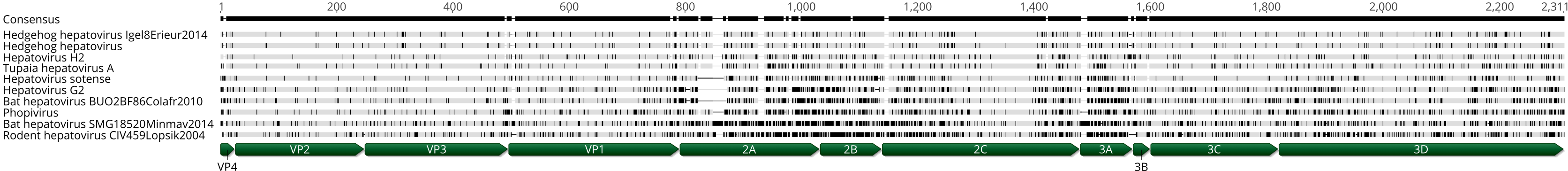

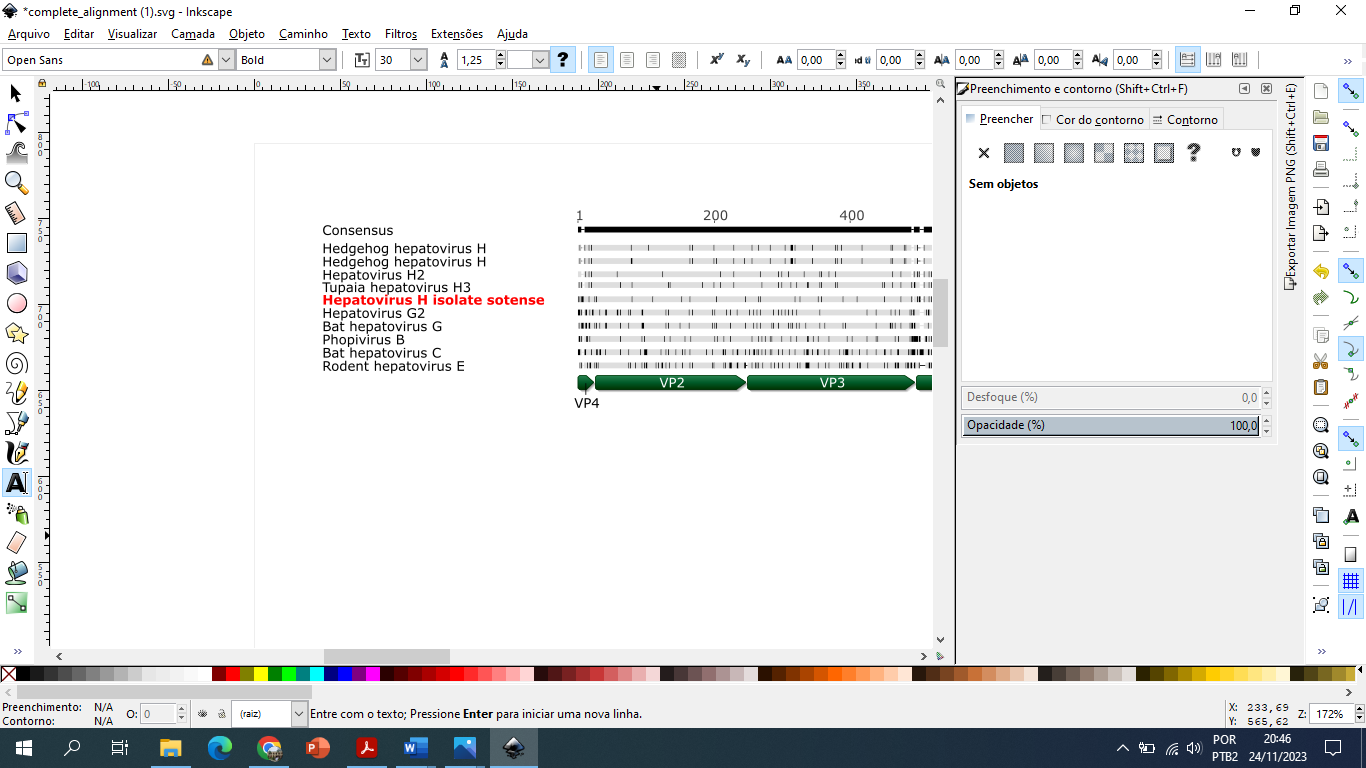

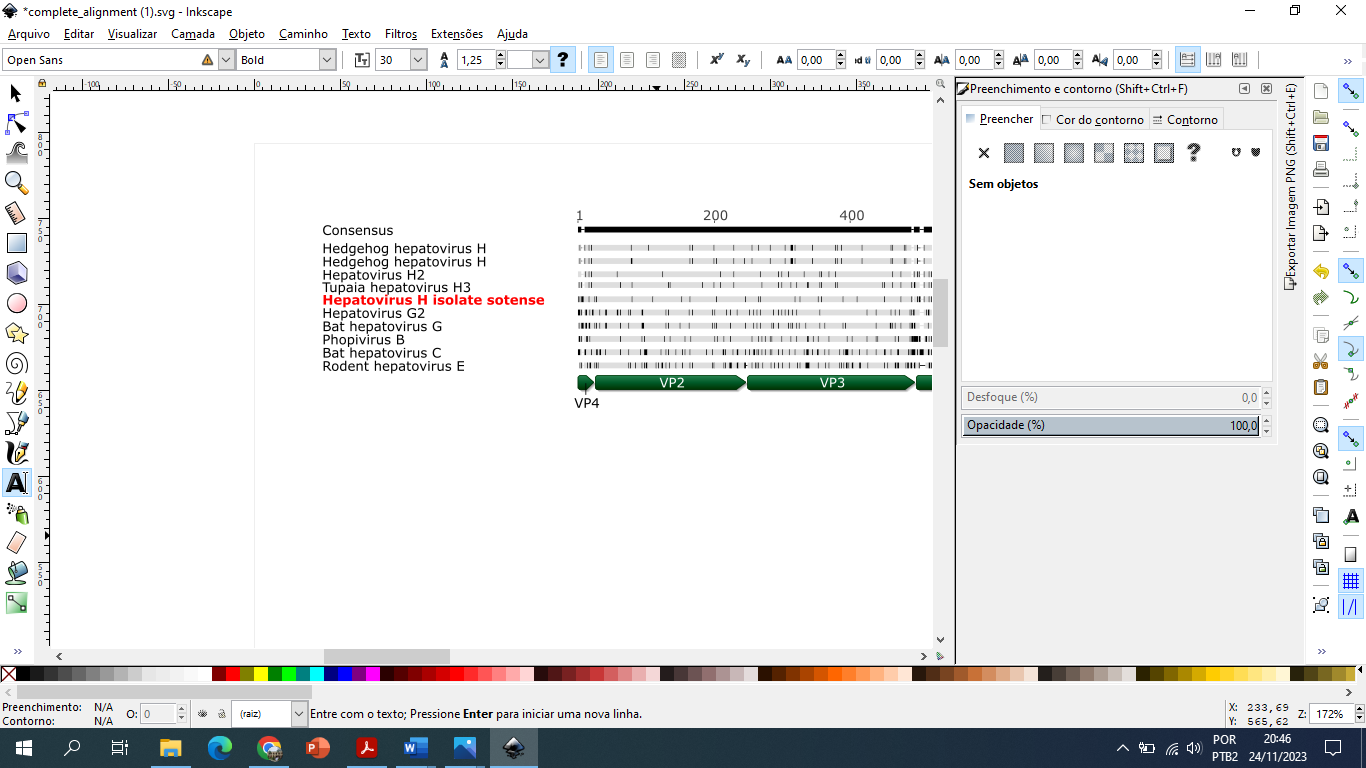

Supplement: Supplementary file 1 — Supplementary Figures. [file 41598_2024_58171_MOESM1_ESM.docx]
